# Standard Immunohistochemical Assays to Assess Autophagy in Mammalian Tissue

**DOI:** 10.3390/cells6030017

**Published:** 2017-06-30

**Authors:** Wim Martinet, Lynn Roth, Guido R. Y. De Meyer

**Affiliations:** Laboratory of Physiopharmacology, University of Antwerp, Universiteitsplein 1, B-2610 Antwerp, Belgium; lynn.roth@uantwerpen.be (L.R.); guido.demeyer@uantwerpen.be (G.R.Y.D.M.)

**Keywords:** autophagy, immunohistochemistry, MAP1LC3, SQSTM1, autophagosome

## Abstract

Autophagy is a highly conserved lysosomal degradation pathway with major impact on diverse human pathologies. Despite the development of different methodologies to detect autophagy both in vitro and in vivo, monitoring autophagy in tissue via immunohistochemical techniques is hampered due to the lack of biomarkers. Immunohistochemical detection of a punctate pattern of ATG8/MAP1LC3 proteins is currently the most frequently used approach to detect autophagy in situ, but it depends on a highly sensitive detection method and is prone to misinterpretation. Moreover, reliable MAP1LC3 immunohistochemical staining requires correct tissue processing and high-quality, isoform-specific antibodies. Immunohistochemical analysis of other autophagy-related protein targets such as SQSTM1, ubiquitin, ATG5 or lysosomal proteins is not recommended as marker for autophagic activity in tissue for multiple reasons including aspecific labeling of cellular structures and a lack of differential protein expression during autophagy initiation. To better understand the role of autophagy in human disease, novel biomarkers for visualization of the autophagic process with standard histology techniques are urgently needed.

## 1. Introduction

**Figure 1:**
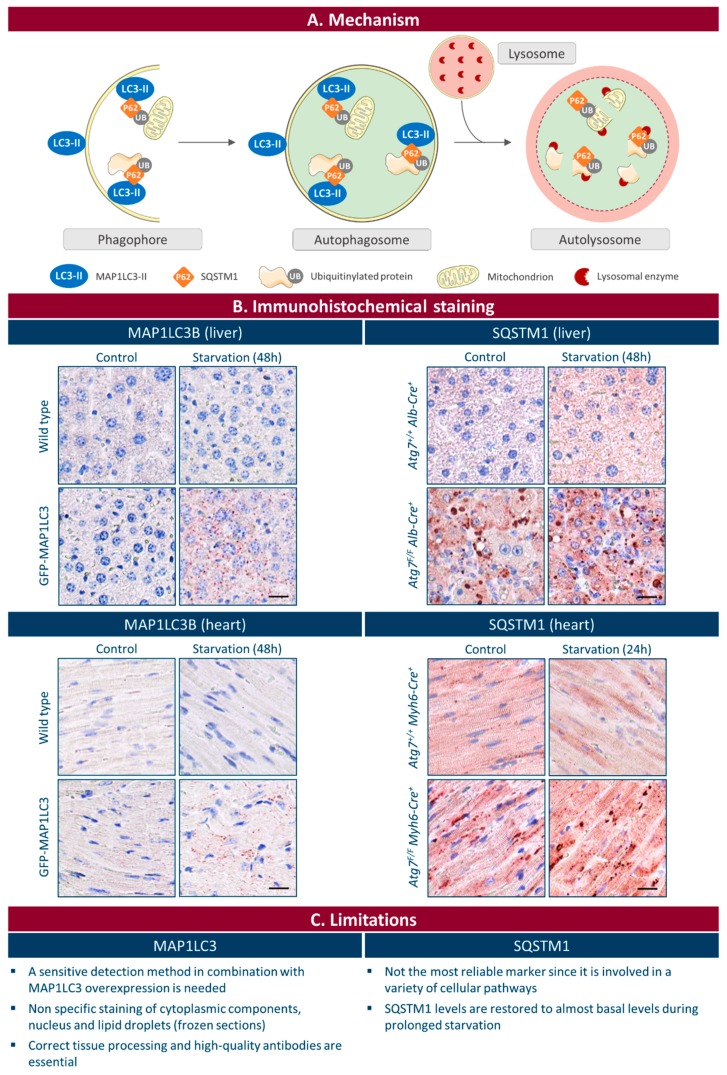
Immunohistochemical detection of MAP1LC3B and SQSTM1 in mammalian tissue. (**A**) Schematic representation of autophagosome formation and the role of MAP1LC3 and SQSTM1. MAP1LC3 plays an essential role in elongation of the phagophore, whereas SQSTM1 serves as an autophagic cargo adaptor. The phagophore forms a vesicular structure called an autophagosome. By fusing with a lysosome, the autophagosome turns into an autolysosome, where the inner membrane as well as its content is broken down by lysosomal enzymes. Thus, autophagy induction is represented by the presence of MAP1LC3-positive puncta on the one hand and by low levels of SQSTM1 on the other hand; (**B**) Representative images of MAP1LC3B and SQSTM1 staining of mouse liver and heart tissue. After fixation in neutral buffered formalin for 24 h, tissues were paraffin-embedded and stained for MAP1LC3B using rabbit monoclonal anti-MAP1LC3B (Cell Signaling, Danvers, MA, USA; 3868) or for SQSTM1 using rabbit polyclonal anti-SQSTM1 (Sigma, Saint Louis, MO, USA; P0067). MAP1LC3-positive puncta are clearly present after starvation (48 h) in GFP-MAP1LC3 transgenic mice. SQSTM1 accumulates in the liver and heart of *Atg7^F/F^ Alb-Cre^+^* (liver-specific deficiency of *Atg7*) and *Atg7^F/F^ Myh6-Cre^+^* mice (cardiac-specific deficiency of *Atg7*), respectively. Scale bar = 20 μm; (**C**) Limitations of MAP1LC3B and SQSTM1 immunohistochemical staining.

Macroautophagy (hereafter referred to as autophagy) is a normal physiological process in the body that maintains intracellular homeostasis by degrading unnecessary or dysfunctional cellular components in lysosomes [1]. The process starts with the formation of a double membrane structure, known as a phagophore or isolation membrane, which may originate from multiple sources including endosomes, the plasma membrane, the Golgi apparatus, mitochondria, and ribosome-free regions of the rough endoplasmic reticulum [2,3,4,5]. The double membrane of a phagophore enwraps small portions of intracellular cytoplasm and forms a vesicular structure called an autophagosome. By fusing with a lysosome, the autophagosome eventually turns into an autolysosome, where the inner membrane of the autophagosome as well as its content is broken down by acid hydrolases (Figure 1A). Because the autophagic process allows recycling of macromolecules and energy, basal autophagy represents a reparative and life-sustaining process that is strictly regulated by a number of highly conserved *AuTophaGy-related* (*ATG*) genes [6].

A large body of evidence indicates that autophagy is involved in the prevention of a wide range of human pathological conditions such as cardiovascular disease [7], neurodegeneration [8], and the initiation of numerous types of cancer [9]. Moreover, autophagy plays a crucial role in embryogenesis [10], aging [11], lipid metabolism [12], vascular reactivity [13], hemostasis [14], as well as maintenance of blood glucose and amino acid levels [15]. While these findings have pressed autophagy to the foreground of scientific research, tissue-based analysis of autophagy remains poorly standardized. Indeed, despite the publication of a third edition of guidelines for monitoring autophagy both in vitro and in vivo [16], demonstration of autophagy in tissue via immunohistochemical techniques has not received extensive evaluation. In this review, we outline the possibilities and limitations of immunohistochemistry for unambiguous detection of autophagy in situ. Given the widespread familiarity and ease of performance, immunohistochemical detection of autophagy-related proteins offers a valuable approach to study autophagy in situ, next to other methodologies such as transmission electron microscopy (TEM) and immunoblotting of tissue lysates.

## 2. Immunohistochemical Detection of ATG8/MAP1LC3

Mammalian autophagy-related 8 (ATG8) proteins are ubiquitin-like proteins that are ubiquitously expressed, although some subfamily members are expressed at increased levels in certain tissues, where they participate in multiple cellular processes such as intracellular membrane trafficking and autophagy [17]. They comprise three subfamilies on the basis of amino acid sequence homology: MAP1LC3 (microtubule-associated protein 1 light chain 3), GABARAP (γ-aminobutyric acid receptor-associated protein), and GATE-16 (Golgi-associated ATPase enhancer of 16 kDa). All three subfamilies are localized to the pre-autophagosomal membrane (phagophore) and are indispensable in the early stages of autophagosome biogenesis. However, it is important to note that they play distinct roles in this process as members of the MAP1LC3 subfamily (for simplicity reasons hereafter referred to as MAP1LC3) are involved in elongation of the phagophore membrane, whereas GABARAP/GATE-16 subfamily members act at a later stage, possibly in sealing of autophagosomes [18]. Moreover, mammalian ATG8 proteins recruit distinct adaptors (e.g., SQSTM1), thereby mediating the delivery of specific cargo (e.g., protein aggregates, organelles) into autophagosomes. Because MAP1LC3 proteins remain bound to the phagosomal membrane after closure and are the best studied and most widely used ATG8 marker proteins to monitor the autophagic process, only this subfamily will be further discussed.

In mammalian cells, at least three different MAP1LC3 isoforms are known: MAP1LC3A, MAP1LC3B, and MAP1LC3C. They are produced as a C-terminally extended precursor, which is rapidly cleaved by the cysteine protease ATG4B to yield the cytosolic form MAP1LC3-I [19,20]. After induction of autophagy, MAP1LC3-I is covalently conjugated to phosphatidylethanolamine (PE) to form MAP1LC3-II via the sequential action of the autophagy proteins ATG7, ATG3, and the ATG12-ATG5-ATG16L1 complex, and finally recruited via PE to the inner and outer surface of autophagosomal membranes (Figure 1A) [19,21]. Accordingly, autophagosomes can be readily identified by immunohistochemistry in tissue sections due to formation of MAP1LC3-positive puncta. In recent years, several protocols have been published for the immunohistochemical detection of MAP1LC3 in formalin fixed paraffin embedded tissue [22,23,24,25,26,27]. Although these protocols offer an elegant way to detect autophagy in tissue, several limitations and/or pitfalls need to be mentioned.

### 2.1. MAP1LC3 Immunohistochemical Staining Depends on a Highly Sensitive Detection Method

In most mammalian tissues—including liver, heart, spleen, and lung—MAP1LC3 is a fragile low-abundance protein making its immunohistochemical detection quite challenging. Fortunately, many tumor cells tend to express higher MAP1LC3 levels as compared to adjacent nonmalignant tissue [23,28,29], but this is certainly not a general phenomenon as decreased expression of MAP1LC3 has been reported in lung carcinomas [30]. A lower expression of MAP1LC3 was also observed in high grade brain carcinomas as compared to lower grade tumors [31]. Moreover, tumors of the same type show heterogeneity in MAP1LC3 staining [23], most likely due to differences in tumor stage and/or intake of anticancer drugs, which are often well-known autophagy agonists. In general, MAP1LC3 staining of noncancerous tissue is weak (or even absent, Figure 1B) so that highly sensitive detection methods combined with MAP1LC3 overexpression are absolutely needed to achieve staining. For instance, it has been reported that tyramide signal amplification is a feasible method to detect MAP1LC3 expression in human tumor xenografts [32]. This technique is based on the conversion of labeled tyramide molecules by horseradish peroxidase (HRP) into highly reactive free radicals that can covalently bind to tyrosine residues near the HRP. Another method, which we recommend, is the use of Envision+ reagent that yields robust MAP1LC3 staining, at least on tissue sections of GFP-MAP1LC3 transgenic mice (Figure 1B) [24,25]. Envision+ allows signal amplification via a hydrophilic dextran polymer, conjugated to secondary antibodies and multiple (up to 100) horseradish peroxidase molecules, and has been used successfully in different studies to identify MAP1LC3 in tissue sections [22,24]. GFP-MAP1LC3 mice are useful to increase sensitivity of MAP1LC3 immunostainings, even though the expression levels of the GFP-MAP1LC3 fusion protein vary considerably among the different organs [33]. Expression of GFP-MAP1LC3 is relatively low in thymus, spleen, and kidney, while high expression levels have been found in heart, liver, pancreas, and skeletal muscle. Also noteworthy is that induction of autophagy (e.g., via nutrient deprivation) is not uniform but organ dependent, meaning that MAP1LC3 puncta can be found in some, but not all tissues of the same GFP-MAP1LC3 mouse regardless of the MAP1LC3 expression level [25,33]. Importantly, MAP1LC3 is rapidly degraded by lysosomal proteases during autophagy, suggesting that MAP1LC3 may not be the most practical immunohistochemical marker to monitor autophagy. Indeed, prolonged autophagy stimulation may actually lead to absence of MAP1LC3 staining rather than abundant staining, unless specific drugs are administered that block the lysosomal degradation of MAP1LC3 (as well as other components in the autophagosome, a process known as autophagic flux). Such drugs include chloroquine (10–100 mg/kg intraperitoneal [IP]) [34,35], leupeptin (9–40 mg/kg IP) [36], or bafilomycin A1 (2.5 mg/kg IP every 12 h) [37] and prevent MAP1LC3 degradation, either via elevation of the lysosomal pH (e.g., chloroquine, bafilomycin A1), which in turn inhibits both fusion of autophagosome with lysosome and lysosomal protein degradation, or via direct inhibition of lysosomal enzymes (e.g., leupeptin). 

### 2.2. MAP1LC3 Immunohistochemical Staining Is Easily Prone to Misinterpretation

For several reasons, immunohistochemical analysis of MAP1LC3 rapidly leads to false conclusions as outlined in more detail below, and thus should be interpreted with caution. Firstly, detection of autophagosomes by immunostaining of MAP1LC3 is technically challenging because of the high background from MAP1LC3-I unassociated with autophagosomes. MAP1LC3-I is diffusely spread in the cell and may yield cytoplasmic staining without clear formation of MAP1LC3-positive puncta. Therefore, diffuse cellular staining may not reflect autophagosome staining, but simply represents MAP1LC3-I protein expression. Granular MAP1LC3 staining is sometimes falsely interpreted as autophagosome formation, but may result from nonspecific staining of cytoplasmic components or from the incorporation of MAP1LC3 into lipofuscin or other lysosomal residual bodies [16]. Secondly, cells with defective autophagy accumulate protein aggregates or cellular structures, in which MAP1LC3 can be incorporated in an autophagy-independent manner [38,39]. Accordingly, large globular intracellular structures may stain positive for MAP1LC3A or MAP1LC3B in autophagy-deficient cells [24]. Similarly, punctate MAP1LC3 staining within nuclei of stressed cells has been reported by several groups [40,41,42], but does not represent autophagosome formation as double-membraned structures cannot be found inside nuclei by TEM analysis [42]. Because MAP1LC3-II is easily detectable in the nucleus [43] and deacetylation of nuclear MAP1LC3 is required for its redistribution to the cytoplasm and subsequent MAP1LC3-ATG7 interaction [44], a likely explanation for nuclear puncta is the formation of MAP1LC3 aggregates and their detection by anti-MAP1LC3 antibodies. Of note, some aspecific puncta are larger than typically seen in the cytoplasm of cells undergoing autophagy. True cytoplasmic puncta are on average 600 nm [45], whereas aspecific puncta are often >1000 nm in diameter. Thirdly, frozen tissue samples are not recommendable for autophagy detection using MAP1LC3 antibodies. Frozen tissue reveals not only poor morphology and resolution, it may also contain lipid droplets that associate with MAP1LC3 proteins. Indeed, previous studies have demonstrated that MAP1LC3 is critically involved in lipid droplet formation and can be localized on the surface of lipid droplets [39,46]. As a consequence, small dot-like MAP1LC3 structures can be visualized around lipid droplets in MAP1LC3 immunostained frozen sections [24]. Finally, even though the best-studied MAP1LC3 isoform for monitoring autophagy is MAP1LC3B, immunoblot experiments show discrete patterns of MAP1LC3A-I and MAP1LC3A-II in various tissues of starved mice, indicating that endogenous MAP1LC3A is also a major marker of autophagic flux [47]. In addition, a transcriptional variant of human MAP1LC3A (variant 1, MAP1LC3Av1) is frequently localized in autophagosomes, similar to MAP1LC3B, and is often inactivated at the transcriptional level in various human cancer cell lines, suggesting that it functions in autophagy and that its inactivation may be crucial for carcinogenesis [48]. However, it is important to note that the immunohistochemical analysis of several tumor types reveals three types of MAP1LC3A expression: diffuse cytoplasmic, perinuclear, and ‘stone-like’ expression [49,50,51]. Diffuse and perinuclear MAP1LC3A staining is observed in both cancerous and noncancerous cells [49], and most likely points to soluble MAP1LC3A that is not associated with autophagosomes. In contrast, ‘stone-like’ intracellular structures represent large clumps (5 µm on average) of amorphous material, typically enclosed within cytoplasmic vacuoles [49,50]. ‘Stone-like’ MAP1LC3A is directly related to high grade tumors, aggressive tumor behavior and poor prognosis [49,50,52,53], and seems to reflect a severe type of autophagy during which autophagic debris accumulates. This debris cannot be further degraded and thus should not be considered as a marker of active autophagy. 

### 2.3. Reliable MAP1LC3 Immunohistochemical Staining Requires Correct Tissue Processing and High-Quality, Isoform-Specific Antibodies

For optimal staining results, tissue samples should be fixed in neutral-buffered formalin (4–10%) for a minimum of 24 h prior to paraffin embedding [22,24]. Samples fixed in the precipitant fixative methacarn or Bouin’s fixative do not stain for MAP1LC3 [24]. The lack of staining in methacarn or Bouin fixed tissue is poorly understood, but could be related to the low molecular weight of MAP1LC3 proteins. Possibly, small proteins such as MAP1LC3 are not large enough to be made insoluble by precipitant fixatives and require sufficient crosslinking to preserve the protein [24]. The optimal buffer during antigen heat retrieval can be either Tris-EDTA (pH 9.0) or citrate buffer (pH 6.0), though we prefer the latter because it yields less background. After antigen retrieval, high-quality antibodies exhibiting minimal cross-reactivity should be employed to achieve a robust and specific immunohistochemical signal (Figure 1B). However, many commercialized antibodies against MAP1LC3B are not isoform-specific and crossreact with MAP1LC3A [24,47]. Recently, Karanasios and Ktistakis (2016) published an extensive list of antibodies for the study of autophagy [54]. This list contains many MAP1LC3 antibodies that are currently used in autophagy assays including immunohistochemistry by autophagy researchers worldwide. Based on experiments in our own laboratory, we recommend the following isoform-specific MAP1LC3 antibodies for immunohistochemical analysis: rabbit polyclonal anti-MAP1LC3A (Abgent, San Diego, CA, USA; AP1805a), rabbit polyclonal anti-MAP1LC3A (Abcam, Cambridge, UK; ab62720), mouse monoclonal anti-MAP1LC3B (clone 5F10; Nanotools, Teningen, Germany; 0231-100/LC3-5F10), and rabbit monoclonal anti-MAP1LC3B (clone D11, Cell Signaling, Danvers, MA, USA; 3868) [24,25]. 

## 3. Immunohistochemical Detection of SQSTM1

Sequestosome 1 (SQSTM1), also known as p62, is a ubiquitously expressed cellular protein that serves as an autophagic cargo adaptor. It is capable of binding to both ubiquitinated and MAP1LC3 proteins, via the ubiquitin-associated (UBA) domain and MAP1LC3-interacting region (LIR), respectively [55]. Because of its interaction with MAP1LC3, SQSTM1 is selectively incorporated in autophagosomes and subsequently degraded (Figure 1A). As a consequence, low levels of SQSTM1 indicates autophagy induction, while accumulation of SQSTM1 aggregates points to inhibition of autophagy (Figure 1B) [56]. Recently, optimized protocols for immunohistochemical analysis of SQSTM1 have been published [26,27,57], which may help to evaluate autophagic flux in a variety of cell types. However, SQSTM1 is not the most reliable marker of autophagic flux as it is involved in a variety of cellular pathways. Accordingly, SQSTM1 expression levels are regulated by a number of factors such as prostate-derived Ets factor (PDEF) [58], the oncogene Ras [59], p38 MAP kinase, and transcription factors Nrf2 and TFEB. In line with these findings, SQSTM1 upregulation is observed during conditions of cellular stress including inflammation and ER stress [60] or during cancer development [58]. Furthermore, experiments with cells in culture have demonstrated that the expression levels of SQSTM1 during starvation-induced autophagy are initially reduced, as expected, due to autophagic degradation, but that SQSTM1 levels are restored to almost basal levels during prolonged starvation [61]. Such restoration depends on transcriptional upregulation of SQSTM1 as well as on the supply of sufficient amounts of intracellular amino acids derived from autophagy that promote de novo synthesis of SQSTM1 [61].

## 4. Immunohistochemical Detection of Other Autophagy-Related Proteins

Polyubiquitinated aggregates of misfolded or damaged proteins in the cytosol are recruited to autophagosomes for subsequent degradation via autophagy and were therefore frequently used in the early 2000s as markers for autophagic cell death during heart failure [62,63] or in calcified aortic valve stenosis [64]. In this light, detailed protocols for immunohistochemical analysis of ubiquitinated aggregates have been published in recent literature [65]. However, given that ubiquitin inclusions colocalize with enhanced levels of SQSTM1 [25], a selective substrate of autophagy that accumulates under autophagy-deficient conditions (vide supra), it is more likely that ubiquitinated aggregates may indicate a block in autophagy rather than autophagic activity. These ubiquitin inclusions may also correspond to inhibition of proteasomal degradation or to structural changes in the substrate proteins that hinder their degradation [16]. Apart from ubiquitin, several other marker proteins such as ULK1 [66], ATG5 [67,68], ATG7 [69], ATG12 [67], cathepsin D [24,65], LAMP1 [65,70], and beclin 1 [67,71] have previously been used to detect autophagy in tissue via immunohistochemistry. However, one caution is that a punctate staining pattern characteristic of autophagosome formation is rarely reported. Another important issue is that the expression of many autophagy-related proteins such as ATG5 or beclin 1 do not significantly change when autophagy is induced, and even if these proteins are differentially expressed, the extent of increase is often cell type- and tissue-dependent [16]. Lysosomal proteins such as cathepsins or LAMP1 are not ideal markers for autophagy because they primarily detect lysosomes and an increase in lysosome size or number could reflect an increase in nonprofessional phagocytosis or general lysosomal activity rather than autophagy [16]. Moreover, the expression pattern of cathepsin D does not necessarily change during starvation-induced autophagy [24]. Finally, it should be noted that nuclear staining of autophagy-related proteins, as previously shown for ATG5 [24] or ubiquitin [72], is usually not considered to be autophagy-specific. Cellular components should be present in the cytosol at all times before they can be degraded via (macro)autophagy. Recent evidence indicates that MAP1LC3 is involved in autophagy-mediated degradation of nuclear lamina B1 [73], but also here nucleus-to-cytoplasm transport of the MAP1LC3-lamina B1 conjugate is a prerequisite for subsequent degradation in lysosomes. 

## 5. Conclusions and Future Perspectives

**Figure 2:**
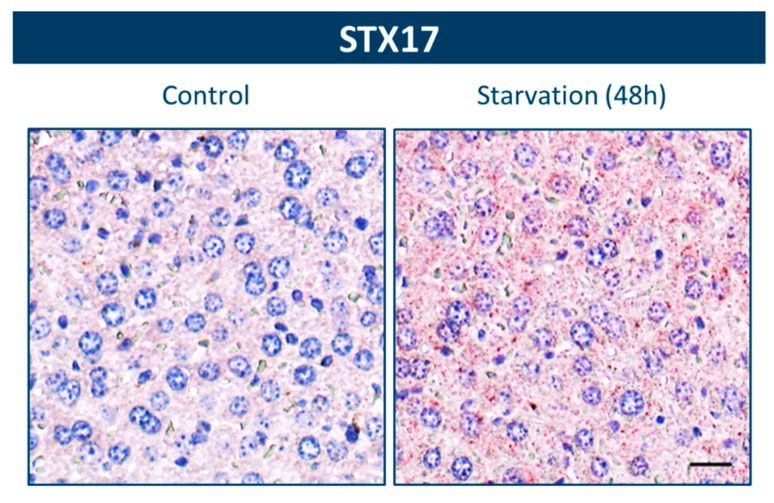
Immunohistochemical detection of STX17 in liver tissue. After fixation in neutral buffered formalin for 24 h, liver samples of wild type mice were paraffin-embedded and stained for STX17 using rabbit polyclonal anti-STX17 (Novus Biologicals, Littleton, CO, USA; NBP1-93968). Heat-mediated antigen retrieval was performed in citrate buffer (pH 6.0). An accumulation of STX17-positive dots is detectable after starvation. Scale bar = 20 μm.

Hitherto, detection of MAP1LC3 punctae remains the most reliable method to monitor autophagy via immunohistochemistry (Figure 1B). However, the broad applicability of this approach is hampered due to low in situ levels of this protein and the easiness by which MAP1LC3 immunostains are misinterpreted (Figure 1C). Immunohistochemical analysis of other autophagy-related protein targets (e.g., ubiquitin, ATG5, lysosomal proteins) may not be trustworthy as marker for autophagic activity in tissue for different reasons. During the latest Keystone Symposium on Autophagy in Whistler, Canada, a debate about autophagy and future directions of the autophagy field was organized. One question raised by a researcher in the audience was: “how can we detect autophagy in tissue?” A panel of experts consisting of Eric Baehrecke (University of Massachusetts Medical School, USA), Ana Maria Cuervo (Albert Einstein College of Medicine, USA), Vojo Deretic (University of New Mexico Health Sciences Center, USA), and Leon Murphy (Novartis Pharmaceuticals, USA) concluded that the options for the unambiguous detection of autophagy in tissue are scarce. Dr. Cuervo suggested that TEM, given its high-resolving power (for reviews see [25,45]), remains the gold standard even today to assess autophagy in tissue. However, because the correct identification of autophagic vacuoles by TEM is challenging [25,74], we definitely need more biomarkers for autophagosome formation. An interesting candidate is ATG9A, a ubiquitously expressed transmembrane protein that acts as a lipid carrier for expansion of the phagophore [75]. Recent studies in yeast indicate that ATG9A expression correlates with the frequency of autophagosome formation and may be used as an early marker of autophagosome assembly [76]. We performed a pilot experiment to detect ATG9A in mouse liver and heart tissue. Unfortunately, we could not obtain a clear autophagosomal (dot-like) staining. Possibly, also ATG9A detection needs highly sensitive methods, analogous with MAP1LC3, and the use of a mouse model that overexpresses ATG9A to achieve optimal staining. Another recently identified protein is syntaxin 17 (STX17), an autophagosomal SNARE that localizes to the outer membrane of completed autophagosomes but not to isolation membranes [77,78]. It interacts with cytosolic SNAP29 and lysosomal VAMP8, thereby mediating the fusion of the autophagosome with a lysosome [79]. We also investigated the potential of this protein as a possible autophagy marker. The staining pattern in the liver of wild type mice looked very promising, with a clear upregulation of STX17-positive dots after starvation (Figure 2). However, a more in-depth analysis is needed to determine whether STX17 staining can be used for the detection of autophagy in other tissues besides the liver. Furthermore, the tissue fixation and staining protocol might play an important role in the sensitivity of this method and should be optimized. In general, to better understand the role of autophagy in many human diseases and to develop therapeutic strategies for clinical management of the autophagic pathway, further validation of STX17 and other proteins as potential biomarkers of autophagy will be essential.

## References

[1] Mizushima N., Komatsu M. (2011). Autophagy: Renovation of cells and tissues. Cell.

[2] Axe E.L., Walker S.A., Manifava M., Chandra P., Roderick H.L., Habermann A., Griffiths G., Ktistakis N.T. (2008). Autophagosome formation from membrane compartments enriched in phosphatidylinositol 3-phosphate and dynamically connected to the endoplasmic reticulum. J. Cell Biol..

[3] Itoh T., Fujita N., Kanno E., Yamamoto A., Yoshimori T., Fukuda M. (2008). Golgi-resident small GTPase Rab33B interacts with ATG16L and modulates autophagosome formation. Mol. Biol. Cell.

[4] Hailey D.W., Rambold A.S., Satpute-Krishnan P., Mitra K., Sougrat R., Kim P.K., Lippincott-Schwartz J. (2010). Mitochondria supply membranes for autophagosome biogenesis during starvation. Cell.

[5] Ravikumar B., Moreau K., Jahreiss L., Puri C., Rubinsztein D.C. (2010). Plasma membrane contributes to the formation of pre-autophagosomal structures. Nat. Cell Biol..

[6] Feng Y., He D., Yao Z., Klionsky D.J. (2014). The machinery of macroautophagy. Cell Res..

[7] Lavandero S., Troncoso R., Rothermel B.A., Martinet W., Sadoshima J., Hill J.A. (2013). Cardiovascular autophagy: Concepts, controversies, and perspectives. Autophagy.

[8] Frake R.A., Ricketts T., Menzies F.M., Rubinsztein D.C. (2015). Autophagy and neurodegeneration. J. Clin. Investig..

[9] White E. (2015). The role for autophagy in cancer. J. Clin. Investig..

[10] Wada Y., Sun-Wada G.H., Kawamura N., Aoyama M. (2014). Role of autophagy in embryogenesis. Curr. Opin. Genet. Dev..

[11] Rubinsztein D.C., Marino G., Kroemer G. (2011). Autophagy and aging. Cell.

[12] Singh R., Kaushik S., Wang Y., Xiang Y., Novak I., Komatsu M., Tanaka K., Cuervo A.M., Czaja M.J. (2009). Autophagy regulates lipid metabolism. Nature.

[13] Michiels C.F., Fransen P., De Munck D.G., De Meyer G.R., Martinet W. (2015). Defective autophagy in vascular smooth muscle cells alters contractility and Ca(2)(+) homeostasis in mice. Am. J. Physiol. Heart Circ. Physiol..

[14] Ouseph M.M., Huang Y., Banerjee M., Joshi S., MacDonald L., Zhong Y., Liu H., Li X., Xiang B., Zhang G. (2015). Autophagy is induced upon platelet activation and is essential for hemostasis and thrombosis. Blood.

[15] Ezaki J., Matsumoto N., Takeda-Ezaki M., Komatsu M., Takahashi K., Hiraoka Y., Taka H., Fujimura T., Takehana K., Yoshida M. (2011). Liver autophagy contributes to the maintenance of blood glucose and amino acid levels. Autophagy.

[16] Klionsky D.J., Abdelmohsen K., Abe A., Abedin M.J., Abeliovich H., Acevedo Arozena A., Adachi H., Adams C.M., Adams P.D., Adeli K. (2016). Guidelines for the use and interpretation of assays for monitoring autophagy (3rd edition). Autophagy.

[17] Shpilka T., Weidberg H., Pietrokovski S., Elazar Z. (2011). Atg8: An autophagy-related ubiquitin-like protein family. Genome Biol..

[18] Weidberg H., Shvets E., Shpilka T., Shimron F., Shinder V., Elazar Z. (2010). LC3 and GATE-16/GABARAP subfamilies are both essential yet act differently in autophagosome biogenesis. EMBO J..

[19] Yoshimori T. (2004). Autophagy: A regulated bulk degradation process inside cells. Biochem. Biophys. Res. Commun..

[20] Li M., Hou Y., Wang J., Chen X., Shao Z.M., Yin X.M. (2011). Kinetics comparisons of mammalian ATG4 homologues indicate selective preferences toward diverse ATG8 substrates. J. Biol. Chem..

[21] Ishibashi K., Fujita N., Kanno E., Omori H., Yoshimori T., Itoh T., Fukuda M. (2011). ATG16L2, a novel isoform of mammalian ATG16L that is not essential for canonical autophagy despite forming an ATG12-5-16l2 complex. Autophagy.

[22] Rosenfeldt M.T., Nixon C., Liu E., Mah L.Y., Ryan K.M. (2012). Analysis of macroautophagy by immunohistochemistry. Autophagy.

[23] Ladoire S., Chaba K., Martins I., Sukkurwala A.Q., Adjemian S., Michaud M., Poirier-Colame V., Andreiuolo F., Galluzzi L., White E. (2012). Immunohistochemical detection of cytoplasmic LC3 puncta in human cancer specimens. Autophagy.

[24] Martinet W., Schrijvers D.M., Timmermans J.P., Bult H., De Meyer G.R. (2013). Immunohistochemical analysis of macroautophagy: Recommendations and limitations. Autophagy.

[25] Martinet W., Timmermans J.P., De Meyer G.R. (2014). Methods to assess autophagy *in situ*–transmission electron microscopy versus immunohistochemistry. Methods Enzymol..

[26] Schlafli A.M., Berezowska S., Adams O., Langer R., Tschan M.P. (2015). Reliable LC3 and p62 autophagy marker detection in formalin fixed paraffin embedded human tissue by immunohistochemistry. Eur. J. Histochem..

[27] Berezowska S., Galvan J.A. (2017). Immunohistochemical detection of the autophagy markers LC3 and p62/SQSTM1 in formalin-fixed and paraffin-embedded tissue. Methods Mol. Biol..

[28] Li X., Lin X., Ma H. (2015). Overexpression of LC3 in papillary thyroid carcinomas and lymph node metastases. Acta Chir. Belg..

[29] Wu S., Sun C., Tian D., Li Y., Gao X., He S., Li T. (2015). Expression and clinical significances of beclin1, LC3 and mtor in colorectal cancer. Int. J. Clin. Exp. Pathol..

[30] Jiang Z.F., Shao L.J., Wang W.M., Yan X.B., Liu R.Y. (2012). Decreased expression of beclin-1 and LC3 in human lung cancer. Mol. Biol. Rep..

[31] Huang X., Bai H.M., Chen L., Li B., Lu Y.C. (2010). Reduced expression of LC3B-II and beclin 1 in glioblastoma multiforme indicates a down-regulated autophagic capacity that relates to the progression of astrocytic tumors. J. Clin. Neurosci..

[32] He H., Yang Y., Xiang Z., Yu L., Chouitar J., Yu J., D’Amore N.R., Li P., Li Z., Bowman D. (2016). A sensitive IHC method for monitoring autophagy-specific markers in human tumor xenografts. J. Biomark.

[33] Mizushima N., Yamamoto A., Matsui M., Yoshimori T., Ohsumi Y. (2004). In vivo analysis of autophagy in response to nutrient starvation using transgenic mice expressing a fluorescent autophagosome marker. Mol. Biol. Cell.

[34] Gurney M.A., Huang C., Ramil J.M., Ravindran N., Andres A.M., Sin J., Linton P.-J., Gottlieb R.A., Mor G., Alvero A.B. (2015). Measuring cardiac autophagic flux *in vitro* and in vivo. Apoptosis and Cancer: Methods and Protocols.

[35] Kurdi A., De Doncker M., Leloup A., Neels H., Timmermans J.P., Lemmens K., Apers S., De Meyer G.R., Martinet W. (2016). Continuous administration of the mTORC1 inhibitor everolimus induces tolerance and decreases autophagy in mice. Br. J. Pharmacol..

[36] Haspel J., Shaik R.S., Ifedigbo E., Nakahira K., Dolinay T., Englert J.A., Choi A.M.K. (2011). Characterization of macroautophagic flux in vivo using a leupeptin-based assay. Autophagy.

[37] Tian Z., Wang C., Hu C., Tian Y., Liu J., Wang X. (2014). Autophagic-lysosomal inhibition compromises ubiquitin-proteasome system performance in a p62 dependent manner in cardiomyocytes. PLoS ONE.

[38] Kuma A., Matsui M., Mizushima N. (2007). LC3, an autophagosome marker, can be incorporated into protein aggregates independent of autophagy: Caution in the interpretation of LC3 localization. Autophagy.

[39] Shibata M., Yoshimura K., Furuya N., Koike M., Ueno T., Komatsu M., Arai H., Tanaka K., Kominami E., Uchiyama Y. (2009). The MAP1-LC3 conjugation system is involved in lipid droplet formation. Biochem. Biophys. Res. Commun..

[40] Martinez-Lopez N., Athonvarangkul D., Mishall P., Sahu S., Singh R. (2013). Autophagy proteins regulate ERK phosphorylation. Nat. Commun..

[41] Corum D.G., Tsichlis P.N., Muise-Helmericks R.C. (2014). Akt3 controls mitochondrial biogenesis and autophagy via regulation of the major nuclear export protein CRM-1. FASEB J..

[42] Buckingham E.M., Carpenter J.E., Jackson W., Grose C. (2014). Nuclear LC3-positive puncta in stressed cells do not represent autophagosomes. Biotechniques.

[43] Karim M.R., Kanazawa T., Daigaku Y., Fujimura S., Miotto G., Kadowaki M. (2007). Cytosolic LC3 ratio as a sensitive index of macroautophagy in isolated rat hepatocytes and H4-II-E cells. Autophagy.

[44] Huang R., Xu Y., Wan W., Shou X., Qian J., You Z., Liu B., Chang C., Zhou T., Lippincott-Schwartz J. (2015). Deacetylation of nuclear LC3 drives autophagy initiation under starvation. Mol. Cell.

[45] Yla-Anttila P., Vihinen H., Jokitalo E., Eskelinen E.L. (2009). Monitoring autophagy by electron microscopy in mammalian cells. Methods Enzymol..

[46] Shibata M., Yoshimura K., Tamura H., Ueno T., Nishimura T., Inoue T., Sasaki M., Koike M., Arai H., Kominami E. (2010). LC3, a microtubule-associated protein1A/B light chain3, is involved in cytoplasmic lipid droplet formation. Biochem. Biophys. Res. Commun..

[47] Zois C.E., Giatromanolaki A., Sivridis E., Papaiakovou M., Kainulainen H., Koukourakis M.I. (2011). “Autophagic flux” in normal mouse tissues: Focus on endogenous LC3A processing. Autophagy.

[48] Bai H., Inoue J., Kawano T., Inazawa J. (2012). A transcriptional variant of the LC3A gene is involved in autophagy and frequently inactivated in human cancers. Oncogene.

[49] Sivridis E., Koukourakis M.I., Zois C.E., Ledaki I., Ferguson D.J., Harris A.L., Gatter K.C., Giatromanolaki A. (2010). LC3A-positive light microscopy detected patterns of autophagy and prognosis in operable breast carcinomas. Am. J. Pathol..

[50] Sivridis E., Giatromanolaki A., Karpathiou G., Karpouzis A., Kouskoukis C., Koukourakis M.I. (2011). LC3A-positive “stone-like” structures in cutaneous squamous cell carcinomas. Am. J. Dermatopathol..

[51] Xi S.Y., Lu J.B., Chen J.W., Cao Y., Luo R.Z., Wu Q.L., Cai M.Y. (2013). The “stone-like” pattern of LC3A expression and its clinicopathologic significance in hepatocellular carcinoma. Biochem. Biophys. Res. Commun..

[52] Giatromanolaki A., Koukourakis M.I., Harris A.L., Polychronidis A., Gatter K.C., Sivridis E. (2010). Prognostic relevance of light chain 3 (LC3A) autophagy patterns in colorectal adenocarcinomas. J. Clin. Pathol..

[53] Karpathiou G., Sivridis E., Koukourakis M.I., Mikroulis D., Bouros D., Froudarakis M.E., Giatromanolaki A. (2011). Light-chain 3A autophagic activity and prognostic significance in non-small cell lung carcinomas. Chest.

[54] Karanasios E., Ktistakis N.T. (2016). Studying autophagy: List of useful antibodies produced via a community effort. Autophagy at the Cell, Tissue and Organismal Level.

[55] Komatsu M., Kageyama S., Ichimura Y. (2012). P62/SQSTM1/A170: Physiology and pathology. Pharmacol. Res..

[56] Komatsu M., Ichimura Y. (2010). Physiological significance of selective degradation of p62 by autophagy. FEBS Lett..

[57] Watson A.S., Soilleux E.J. (2015). Detection of p62 on paraffin sections by immunohistochemistry. Cold Spring Harb. Protoc..

[58] Thompson H.G., Harris J.W., Wold B.J., Lin F., Brody J.P. (2003). P62 overexpression in breast tumors and regulation by prostate-derived ets factor in breast cancer cells. Oncogene.

[59] Duran A., Linares J.F., Galvez A.S., Wikenheiser K., Flores J.M., Diaz-Meco M.T., Moscat J. (2008). The signaling adaptor p62 is an important NF-kappaB mediator in tumorigenesis. Cancer Cell.

[60] Liu X.D., Ko S., Xu Y., Fattah E.A., Xiang Q., Jagannath C., Ishii T., Komatsu M., Eissa N.T. (2012). Transient aggregation of ubiquitinated proteins is a cytosolic unfolded protein response to inflammation and endoplasmic reticulum stress. J. Biol. Chem..

[61] Sahani M.H., Itakura E., Mizushima N. (2014). Expression of the autophagy substrate SQSTM1/p62 is restored during prolonged starvation depending on transcriptional upregulation and autophagy-derived amino acids. Autophagy.

[62] Knaapen M.W., Davies M.J., De Bie M., Haven A.J., Martinet W., Kockx M.M. (2001). Apoptotic versus autophagic cell death in heart failure. Cardiovasc. Res..

[63] Kostin S., Pool L., Elsasser A., Hein S., Drexler H.C., Arnon E., Hayakawa Y., Zimmermann R., Bauer E., Klovekorn W.P. (2003). Myocytes die by multiple mechanisms in failing human hearts. Circ. Res..

[64] Somers P., Knaapen M., Kockx M., van Cauwelaert P., Bortier H., Mistiaen W. (2006). Histological evaluation of autophagic cell death in calcified aortic valve stenosis. J. Heart Valve Dis..

[65] Zhu H., Rothermel B.A., Hill J.A. (2009). Autophagy in load-induced heart disease. Methods Enzymol..

[66] Yun M., Bai H.Y., Zhang J.X., Rong J., Weng H.W., Zheng Z.S., Xu Y., Tong Z.T., Huang X.X., Liao Y.J. (2015). ULK1: A promising biomarker in predicting poor prognosis and therapeutic response in human nasopharygeal carcinoma. PLoS ONE.

[67] Ma J.F., Huang Y., Chen S.D., Halliday G. (2010). Immunohistochemical evidence for macroautophagy in neurones and endothelial cells in alzheimer’s disease. Neuropathol. Appl. Neurobiol..

[68] Vigen R.A., Kodama Y., Viset T., Fossmark R., Waldum H., Kidd M., Wang T.C., Modlin I.M., Chen D., Zhao C.M. (2013). Immunohistochemical evidence for an impairment of autophagy in tumorigenesis of gastric carcinoids and adenocarcinomas in rodent models and patients. Histol. Histopathol..

[69] Okada M., Oikawa M., Miki Y., Shimizu Y., Echigo S., Takahashi T., Kumamoto H. (2014). Immunohistochemical assessment of ATG7, LC3, and p62 in ameloblastomas. J. Oral Pathol. Med..

[70] Akazawa H., Komazaki S., Shimomura H., Terasaki F., Zou Y., Takano H., Nagai T., Komuro I. (2004). Diphtheria toxin-induced autophagic cardiomyocyte death plays a pathogenic role in mouse model of heart failure. J. Biol. Chem..

[71] Carloni S., Buonocore G., Balduini W. (2008). Protective role of autophagy in neonatal hypoxia-ischemia induced brain injury. Neurobiol. Dis..

[72] Von Mikecz A., Chen M., Rockel T., Scharf A. (2008). The nuclear ubiquitin-proteasome system: Visualization of proteasomes, protein aggregates, and proteolysis in the cell nucleus. Methods Mol. Biol..

[73] Dou Z., Xu C., Donahue G., Shimi T., Pan J.A., Zhu J., Ivanov A., Capell B.C., Drake A.M., Shah P.P. (2015). Autophagy mediates degradation of nuclear lamina. Nature.

[74] Eskelinen E.L. (2008). To be or not to be? Examples of incorrect identification of autophagic compartments in conventional transmission electron microscopy of mammalian cells. Autophagy.

[75] Tamura H., Shibata M., Koike M., Sasaki M., Uchiyama Y. (2010). ATG9A protein, an autophagy-related membrane protein, is localized in the neurons of mouse brains. J. Histochem. Cytochem..

[76] Jin M., He D., Backues S.K., Freeberg M.A., Liu X., Kim J.K., Klionsky D.J. (2014). Transcriptional regulation by pho23 modulates the frequency of autophagosome formation. Curr. Biol..

[77] Itakura E., Kishi-Itakura C., Mizushima N. (2012). The hairpin-type tail-anchored snare syntaxin 17 targets to autophagosomes for fusion with endosomes/lysosomes. Cell.

[78] Hamasaki M., Furuta N., Matsuda A., Nezu A., Yamamoto A., Fujita N., Oomori H., Noda T., Haraguchi T., Hiraoka Y. (2013). Autophagosomes form at er-mitochondria contact sites. Nature.

[79] Itakura E., Mizushima N. (2013). Syntaxin 17: The autophagosomal snare. Autophagy.

